# Ninth International Congress

**Published:** 1886-01

**Authors:** 


### NINTH INTERNATIONAL MEDICAL CONGRESS.

The primary organization of the Ninth Medical Congress, to be held in Washington, D. C., in September, 1887, is announced by the executive committee, so far as the names of the general officers who will be nominated for election by the Congress go, together with the names of the committeemen; and the rules of the Congress, printed in English, French and German. The Presidents, Vice Presidents, Secretaries and members of councils for each section will be given in full programme, to be published at a later period in the progress of the work.

Meantime, the executive committee ''cordially invites members of the medical profession, and men eminent in the sciences collateral to medicine, in all countries, to participate in the International Congress of 1887.''

